# Large Cell Neuroendocrine Carcinoma of the Lung: A Case Series of 14 Cases

**DOI:** 10.7759/cureus.27559

**Published:** 2022-08-01

**Authors:** Amine Hayoune, Imane Mahfoud, Afaf Thouil, Hatim Kouismi

**Affiliations:** 1 Department of Respiratory Diseases, Faculty of Medicine and Pharmacy of Oujda, Mohammed VI University Hospital, Mohamed First University, Oujda, MAR; 2 Department of Respiratory Diseases, Research and Medical Sciences Laboratory, Faculty of Medicine and Pharmacy of Oujda, Mohammed VI University Hospital, Mohamed First University, Oujda, MAR

**Keywords:** chemotherapy, bronchoscopy, large cell neuroendocrine, tobacco cessation, lung cancer

## Abstract

Pulmonary large cell neuroendocrine carcinoma (LCNEC) is a rare subtype of neuroendocrine tumor, presenting with very aggressive behavior and a poor prognosis. The diagnosis is difficult and requires histological confirmation of the neuroendocrine nature by an immunohistochemical study on a biopsy sample. We retrospectively studied a series of 14 patients from the pneumology department of the Mohammed VI University Hospital of Oujda, Morocco, over a period of five years (from April 2017 to March 2021). The average age was 63.41 years (45-80 years). All our patients were male and smokers. The clinical signs were dominated by deterioration in general condition and dyspnea. Bronchoscopy was performed in 92% of patients, with neoplastic stenosis being the main found aspect, in 35% of cases. The histological diagnosis was obtained by bronchoscopy in 50% of cases. In the remaining cases, it was carried by CT-guided transparietal biopsy in 28% of cases, pleural biopsy in 7% of cases, biopsy of a metastatic site in 7% of cases and finally thoracoscopy with pleural biopsy in the remaining 7% of cases. Therapeutically, no patient received surgical treatment and three patients were put on palliative treatment. The positive diagnosis is often late, which makes the prognosis bad and the therapeutic possibilities limited. Hence the importance of strategies for the prevention of tobacco control and early detection in population at risk.

## Introduction

Neuroendocrine tumors of the lung are a heterogeneous group of cancers that represent about 20% of all lung cancers [1]. They are rare tumors that have as origin the diffuse endocrine system. Among these neoplasms, large cell neuroendocrine carcinoma (LCNEC) is a rare and aggressive malignant tumor, which presents often in the metastatic stage at the time of diagnosis.

## Materials and methods

Our study is descriptive and retrospective, about patients who were diagnosed with lung LCNEC in the pulmonology department of the Mohammed VI University Hospital in Oujda, Morocco. We have included all cases of LCNEC of the lung diagnosed in our department from April 17, 2017, to March 18, 2021. We have excluded cases with incomplete data and who were diagnosed with lung LCNEC in other sanitary structures other than the pulmonology department of the Mohammed VI University Hospital in Oujda, Morocco.

Data collection

All the patients had a complete clinical examination and a thoracic computed tomography (CT). Bronchoscopy was performed in 13 patients. As part of the extension assessment, an abdominopelvic CT and a cerebral CT scan were performed in all cases. To study the exact location of the tumors as well as their sizes, we relied on imaging data. The diagnosis was confirmed by pathological assessment in all cases carried on bronchoscopy with bronchial biopsy (seven cases), a CT-guided lung biopsy (seven cases), a pleural biopsy (one case), a thoracoscopy with pleural biopsy (one case), and a biopsy of a right parasternal swelling (one case). All our samples were fixed in formalin then embedded in paraffin. An immunohistochemical study was performed in all cases.

During our study, we carried out a detailed analysis of our series according to age, sex, circumstances of discovery, radiological presentation, endoscopic and histological aspect, treatment, and different evolutionary modalities.

## Results

Epidemiology and clinical features

The average age of our cases was 63.41 years, with ages ranging from 45 to 80 years. A peak frequency was observed between 60 and 70 years. All patients were male. Smoking was found in all patients and 78.5% (11) of patients were heavy smokers with more than 40 pack-years. Six of them consumed alcohol and three cannabis. Medical history investigations revealed in eight cases, diabetes, hypertension, pulmonary tuberculosis, chronic bronchitis, and poorly controlled asthma with salbutamol. We have also noticed a history of appendectomy in one patient. All our patients were symptomatic at the time of diagnosis, with alteration of the general state and the fever in all cases. Dyspnea was present in 71% of the cases, thoracic pain in 35% of cases, cough and hemoptysis in 21% of cases, and cervicofacial edema in two patients. Only one patient presented with neurological symptoms with gait disorder related to cerebral metastasis with associated signs of intracranial hypertension. The WHO performance status was 4 in three cases, 3 in a single patient, 2 in seven patients, and 1 in three patients. The pleuropulmonary clinical examination revealed digital clubbing, a pleural effusion syndrome in three cases, and superior vena cava syndrome in two cases.

Complementary investigations

**Figure 1:**
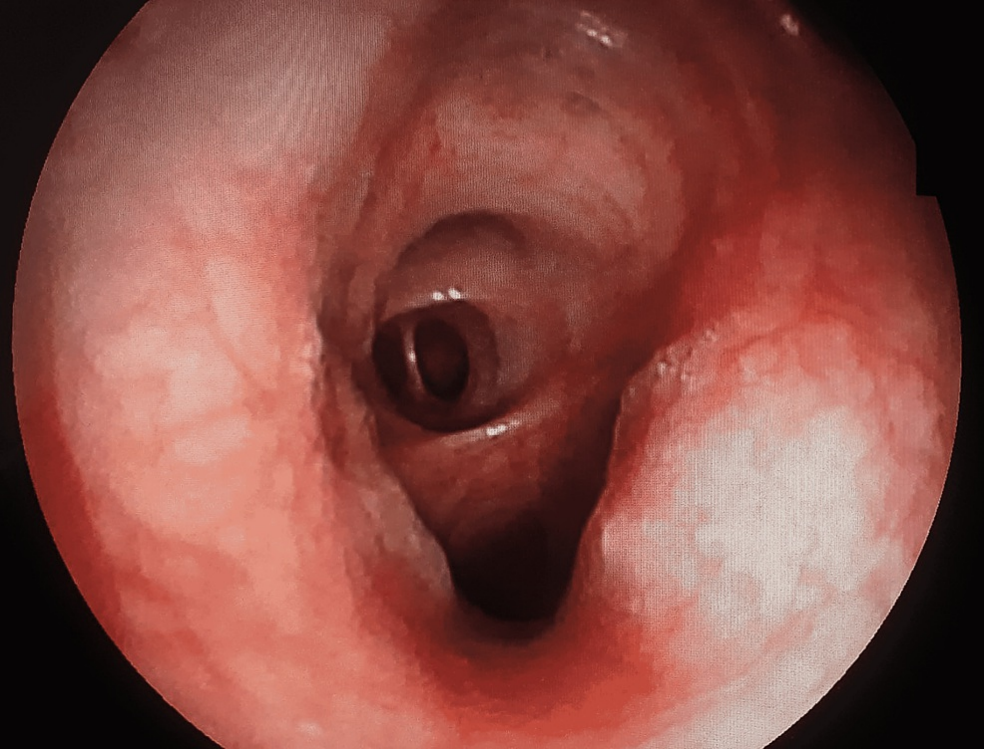
Bronchoscopic photography showing an infiltrative and slightly exophytic appearance of the bronchial mucosa

Thoracic imaging revealed a mass associated with mediastinal lymphadenopathy in all patients, pleural effusion in three cases, a “lâcher de ballons” appearance in one patient, pleural thickening in one patient, and compression and superior vena cava thrombus in one patient. The extension assessment found secondary bone lesions in five patients, lung metastases in four patients, cerebral metastases in three cases, and adrenal hypertrophy in two cases. Bronchoscopy was performed in 13 patients with tumors completely obstructing the airway (35%) (Figure 1); budding and diffuse inflammatory thickening of bronchial spurs in four patients (28%), and a normal bronchoscopic examination in a single patient. Only one patient did not undergo a bronchoscopy whose histological diagnosis was obtained by a pleural biopsy.

Pathological assessment

**Figure 2:**
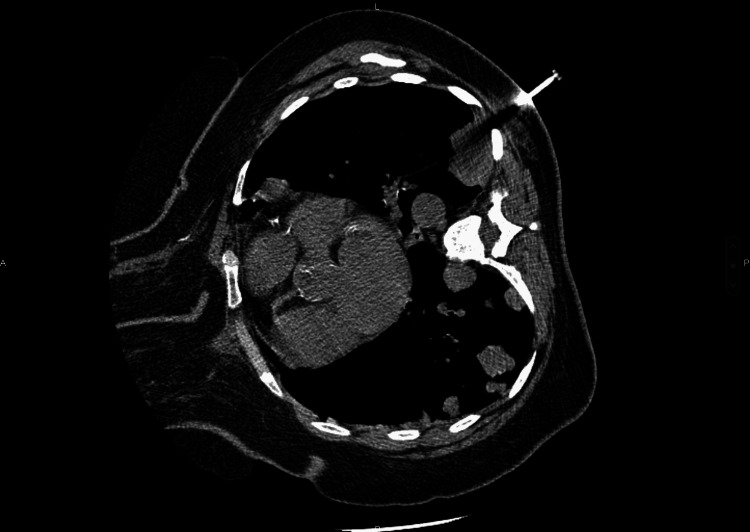
CT-scan guided trans parietal biopsy of a lung mass, revealed to be a lung large cell neuroendocrine carcinoma on pathological assessment

**Figure 3:**
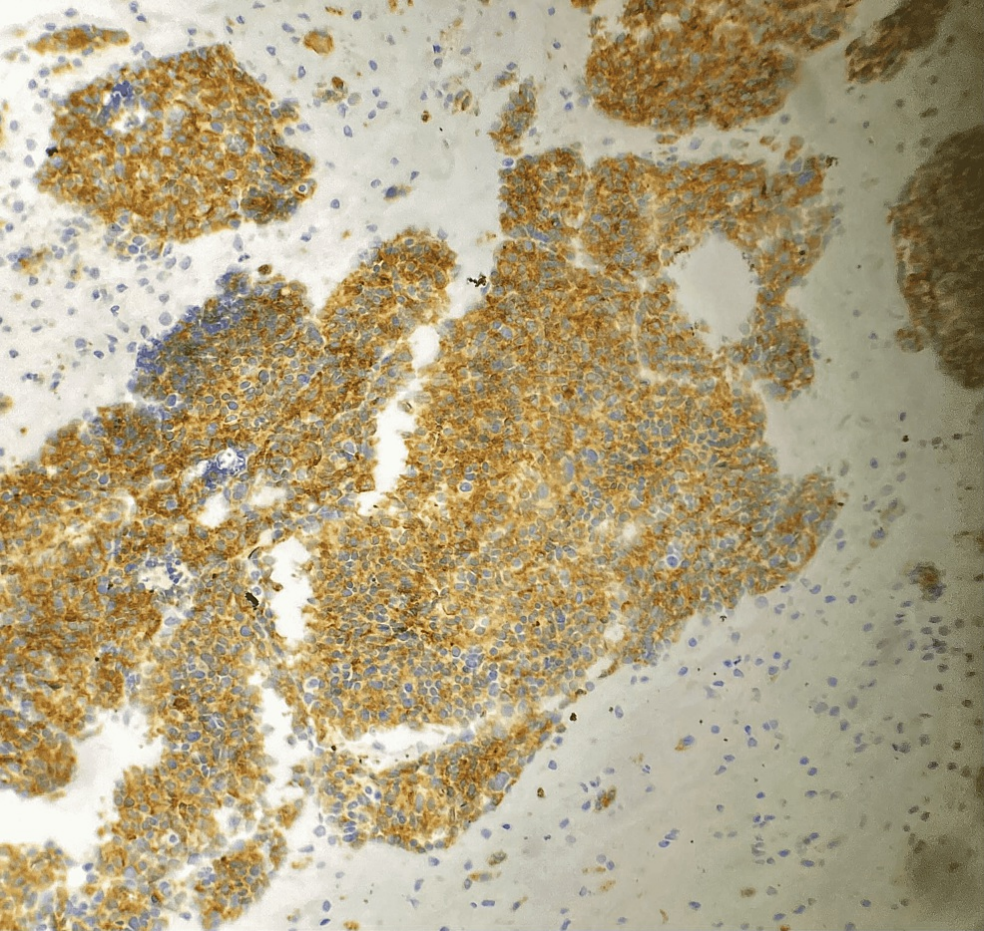
Photomicrograph showing diffuse expression of chromogranin A in neoplastic cells

**Figure 4:**
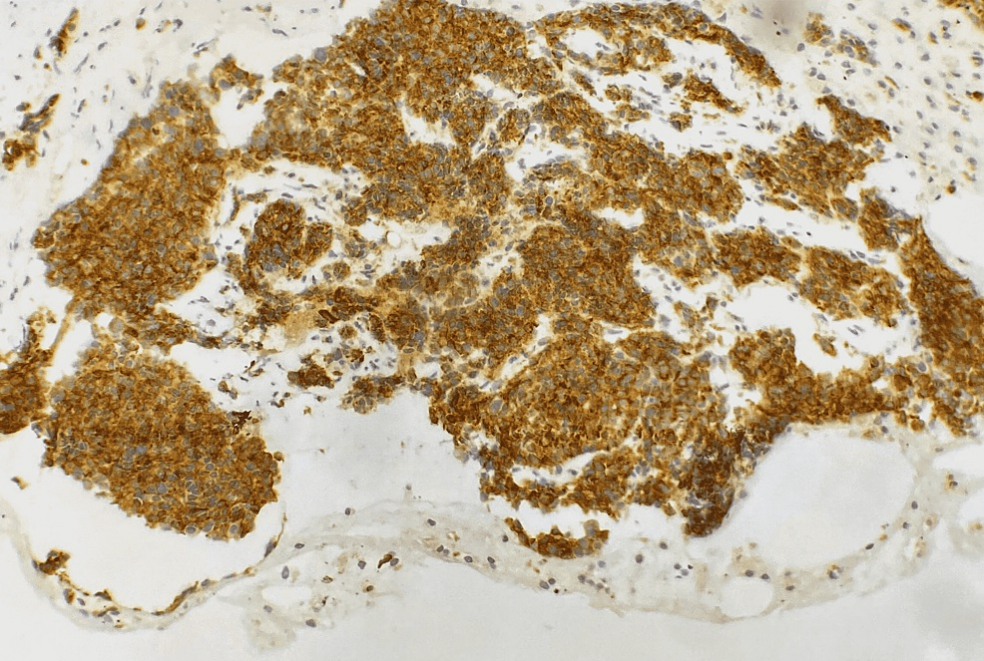
Photomicrograph showing diffuse expression of synaptophysin in neoplastic cells

**Figure 5:**
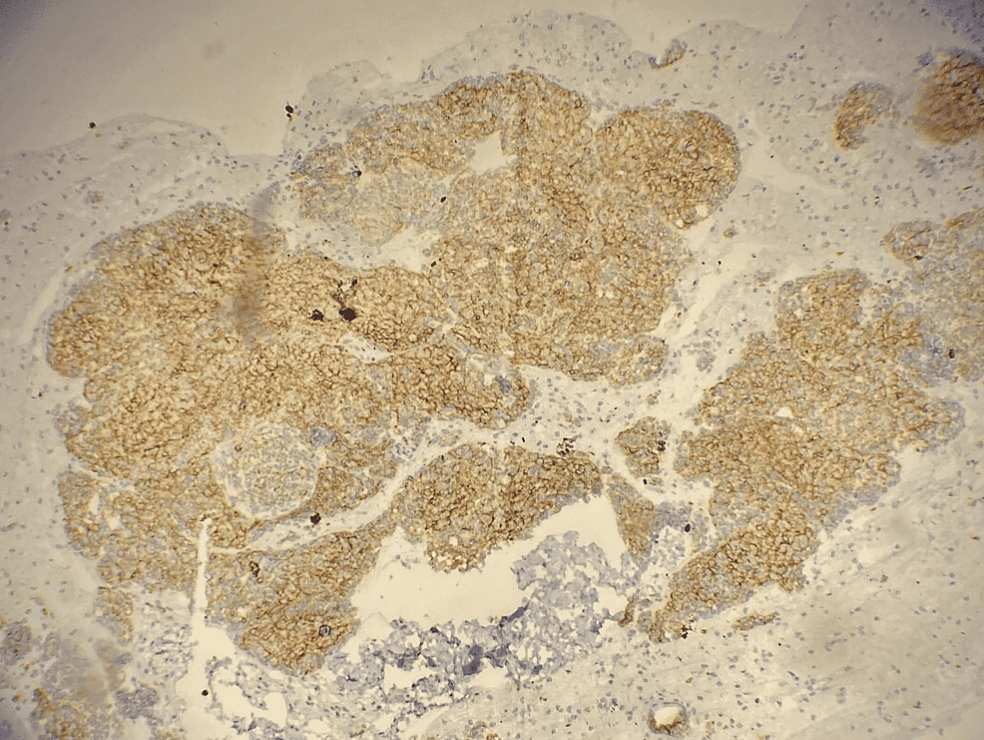
Photomicrograph showing diffuse expression of CD56 in neoplastic cells

Histological confirmation was obtained by assessment of endobronchial biopsies in 50% of cases, CT-guided transparietal biopsy of the mass in 28% of patients (Figure 2), pleural biopsy in one case (7%), biopsy of a right parasternal swelling by parietal tumor extension in one case (7%), and a thoracoscopy with pleural biopsy in one case (7%). The pathological assessment completed by an immunohistochemical study enabled the final diagnosis of LCNEC (Figures 3, 4, 5).

Treatment

After discussion at the multidisciplinary consultation meeting, no patient was deemed to be a surgical candidate. The decision was made to refer our patients to oncology for chemotherapy, the patients who presented with cerebral metastases underwent cerebral radiotherapy, three patients were placed on palliative treatment given their age and a WHO performance status at 4.

## Discussion

Pulmonary LCNECs are rare tumors; in a series of resected lung specimens, the incidence of pulmonary LCNECs seemed to be between 2.1 and 3.5%. Nonetheless, the frequency is higher than approximated due to diagnostic difficulties on cytological specimens: LCNECs contain features of both small cell and non-small cell lung cancer [2]. Unlike typical and atypical carcinoid tumors, LCNECs are generally associated with male gender [3], advanced age, and excessive smoking [4,5]. In our series, all patients were males, with an average age of 63.41 years. The factors involved in the etiology of these tumors are similar to those involved in the other histological types, smoking being the major factor incriminated in more than 90% of cases. Moreover, exposure to asbestos or a history of radiation therapy has also been reported.

LCNECs do not have any specific clinical signs. Affected patients often present with persistent respiratory symptoms (cough, dyspnea, hemoptysis, etc.), in particular in a smoker or former smoker patients. Other signs may also reveal symptoms related to the presence of one or more metastases (Most frequently reported sites are liver, hepatic, bone, and brain) frequently [6,7]. An unexplained change in the performance status, a thromboembolic disease without predisposing circumstances, superior vena cava syndrome, or Pancoast Tobias syndrome in the case of apical location of the tumor. Paraneoplastic syndromes are quite rare at the time of diagnosis. Approximately 25% of affected patients may be asymptomatic [8].

These tumors present on imaging as well-demarcated oval to round masses with lobulated margins. Their size usually varies from 2-5 cm. Internal calcifications are rare (9-21%). A slight proximal predilection may be present [7].

There may be homogeneous or inhomogeneous enhancement after injection of contrast product. Invasion of the chest wall or pleura is common [9].

In all cases, the diagnosis of bronchopulmonary cancer is based on the histological examination of a sample of tumor tissue. Samples are taken from the tumor and/or associated lymphadenopathies, from an accessible metastatic site, or from a pleural effusion. Morphological, immunophenotypic, cytogenetic, and molecular studies make it possible to make a definitive diagnosis of LCNEC. Performing molecular testing of targetable oncogenic drivers such as anaplastic lymphoma kinase (ALK) and estimated glomerular filtration rate (eGFR) alteration is recommended since it guides the management of patients with potential response to targeted therapies. However, testing of RB1/p53 in routine practice is not indicated.

Since Travis et al. proposed that LCNEC is a pulmonary neuroendocrine tumor in 1991 [10], the diagnosis of LCNEC has been established by histological and cytological evaluations, immunohistochemistry, or electron microscopy.

In 2015, the WHO proposed histopathological diagnostic criteria for LCNEC, which include the presence of neuroendocrine architecture (nests, trabeculae, rosettes, palisades), a high mitotic index (≥ 11 mitoses per 2 mm2 (10 HPF) with an average of 70 per 2 mm2 (10 HPF)), presence of necrosis, often in form of large areas, presence of large tumor cells with moderate to abundant cytoplasm, prominent nucleolus, and finally on the immunohistochemical level, one or more positive neuroendocrine markers in immunohistochemistry: synaptophysin, chromogranin, and CD56. A marker is considered to be positive when more than 50% of tumor cells are marked.

Pre-therapeutic clinical assessment

It has no specificity and applies in current practice to diagnostic, fibroscopic, and radiological practices of non-small cell bronchial carcinomas. Performing a cerebral MRI or a cerebral CT scan is recommended. Bone scintigraphy is recommended in case of clinical warning signs. In operable forms, performing a positron emission tomography (PET)/CT is recommended. The use of endobronchial ultrasound (EBUS) is possible for staging [11].

Clinical classification

The one used in daily practice is the TNM (tumor, nodes, and metastases) classification system.

Place of Tumor Markers in the Staging Assessment

Given the low sensitivities and specificities of the serum markers used in terms of lung cancer, they have no utility outside of prospective clinical trials. [12].

Therapeutic care

No standard treatment exists for lung LCNECs. Only a few models are available, mainly from case series studies. Since lung LCNEC is very rare, randomized trials are challenging to carry out.

Five-year survival remains low, despite multimodal treatment in the late stages, and the recurrence incidence after surgery is important [13]. The management of early-stage LCNECs (TNM stages I and II) is not different than that of non-small cell lung cancers, relying above all on surgical excision. This approach is also the primary means of obtaining a histological diagnosis. Unfortunately, lung LCNECs are most often not eligible for surgical resection due to local or systemic dissemination. In the initial stages, the preferred choices are lobectomy or pneumonectomy since they can improve survival if lymph node metastases are absent [13]. Perioperative, neoadjuvant, or adjuvant chemotherapy may be a valid option to prevent cancer relapse [14,15]. A retrospective analysis of 144 surgically resected lung LCNECs revealed better outcomes with preoperative or postoperative chemotherapy in stage I disease. This suggests a promising role of adjuvant therapy in early diagnosed cases [16]. The role of radiotherapy in the treatment of local or advanced pulmonary LCNEC is not yet clear, but some authors suggest its use in the setting of locally advanced disease. There is no consensus on the standard treatment of recurrent or advanced cases of LCNEC [17,18]. In 2005, in a study by Rossi et al., 83 cases of lung LCNEC (65% metastatic patients) were analyzed, aiming to explore clinical and therapeutic histories and to identify new potential therapeutic targets and better strategies for treatment by performing immunohistochemical screening for several tyrosine kinase receptors [19].

Assessment of clinical characteristics confirmed the prevalence of male gender, heavy smoking, the incidence of median age, and occurrence of lung injury in peripheral lung. The main sites of metastases were the brain, liver, and bone. The analysis showed evidence of greater efficacy of platinum-etoposide-based chemotherapy in metastatic patients. The response rate (RR) was at 29%, with complete response (CR) in two cases and four cases of partial responses (PR); in contrast, no CR or PR has been identified in metastatic patients treated with different chemotherapeutic regimens. There is increasing evidence that LCNECs tend to share several characteristics with small cell carcinomas, particularly in terms of response to chemotherapy [20]. Therefore, efforts are made to clarify whether pulmonary LCNECs can be treated as small cell carcinomas, non-small cell carcinomas, or as another lung tumor variant.

The prognosis of LCNECs is poor and this tumor behaves in a biologically aggressive manner, like small cell carcinomas. Stage by stage, the survival curves of pulmonary LCNECs and small cell carcinomas overlap. Moreover, survival is lower than that of other non-small cell carcinomas. Even in patients with possibly resectable stage I lung cancer, the prognosis is poor. The five-year survival rates range from 27% to 67% [18]. For all stages, Iyoda et al. found a five-year survival rate of 35.3% and a five-year disease-free survival rate of 27.4%; a large proportion of relapses occurred during the first two years of follow-up [18,21]. One of the causes of this dramatic situation is the development of second primary, synchronous or metachronous cancers.

Limitation

The limitations of our study include its retrospective nature, the small number of cases in our series, and the unavailability of follow-up data in a large number of patients. A prospective study with long-term patient follow-up is therefore needed.

## Conclusions

Pulmonary LCNEC is a rare subtype of neuroendocrine tumors. The diagnosis of these tumors requires pathological assessment with the often need to use immunohistochemical study on tissue samples. Our study confirms the fact that LCNEC has very aggressive behavior and a poor prognosis. This is further worsened by the often-late diagnosis. As a consequence, therapeutic possibilities are limited. It is important to remind the major role of preventive strategies and early detection in populations at risk.

.
